# Associations of physical activity, disordered eating, and depressive symptoms with academic performance among Saudi university students

**DOI:** 10.3389/fpubh.2026.1769363

**Published:** 2026-02-12

**Authors:** Madawi Alotaibi, Farah Alghamdi, Nourah Almousa, Roaa Almalkia, Reema Alfaifi, Hisaah Alturki, Hathil Alkahal, Ruba Asiri, Muzun Almuzaini, Ghaday Alshahrani, Samiah Alqabbani, Afrah Almuwais, Wafa Alahmari

**Affiliations:** 1 Department of Rehabilitation Sciences, College of Health and Rehabilitation Sciences, Princess Nourah Bint Abdulrahman University, Riyadh, Saudi Arabia; 2 Department of Health Sciences, College of Health and Rehabilitation Sciences, Princess Nourah bint Abdulrahman University, Riyadh, Saudi Arabia; 3 Public Health Program, King Saud bin Abdulaziz University for Health Sciences (KSAU-HS), Riyadh, Saudi Arabia

**Keywords:** academic performance, depression, eating disorders, GPA, lifestyle behaviors, mental health, physical activity

## Abstract

**Background and objectives:**

Academic performance is shaped by various behavioral and psychological factors. However, the effects of physical activity, depressive symptoms, and disordered eating on academic success, particularly among university students in Saudi Arabia, remain underexplored. This study investigates the individual and combined associations of these factors on GPA among Saudi university students.

**Materials and methods:**

A cross-sectional survey was conducted from December 2024 to June 2025 involving a non-probability convenience sample of 400 students from various Saudi universities. Data were collected using validated instruments, including the International Physical Activity Questionnaire–Short Form (IPAQ-SF), the Patient Health Questionnaire-9 (PHQ-9) for assessing depression, and the Eating Disorder Examination Questionnaire–Short Form (EDE-QS). Descriptive statistics, chi-square tests, and multiple linear regression analyses were utilized to identify associations and predictors of academic performance.

**Results:**

The majority of participants were female (79.8%) with a median age of 21. More than half (53%) reported low levels of physical activity, and 50.7% exhibited clinically significant depressive symptoms. Elevated behavioral symptoms of disordered eating, assessed using the behavioral subscale of the EDE-QS, were commonly observed among participants. Regression analysis showed that adherence to WHO-recommended physical activity levels, enrolment in medical disciplines, and lower behavioral symptoms of eating disorders were associated with higher GPA. In contrast, depression and cognitive eating concerns were not meaningfully associated with academic performance.

**Conclusion:**

Physical activity and disordered eating behaviors play a crucial role in influencing academic performance among Saudi university students, while depression, despite its high prevalence, did not serve as an independent predictor of GPA. These findings highlight the necessity of implementing integrated health promotion strategies within academic institutions that address both mental health and lifestyle behaviors to enhance student success.

## 1 Introduction

Academic performance is a critical component of student success in higher education, with far-reaching implications for future employment, career progression, and socioeconomic mobility. In the Kingdom of Saudi Arabia (KSA), student achievement is largely assessed through Grade Point Average (GPA), which serves as an essential criterion for access to competitive internships, scholarships, and graduate programs (1).

However, relying solely on GPA fails to capture the multifaceted nature of academic success, which is influenced by a variety of interrelated factors, such as physical health, mental well-being, and lifestyle choices. Based on the biopsychosocial model, academic performance is shaped by the dynamic interaction of biological, psychological, and behavioral factors (2). Within this framework, elements such as physical activity, depressive symptoms, and the risk of eating disorders emerge as significant yet underexplored contributors to students’ academic functioning.

Physical activity is widely acknowledged for its essential role in enhancing cognitive functioning, emotional regulation, and overall health—factors that contribute significantly to academic success. According to the Physical Activity Statistics Publication (3), 58.5% of the Saudi population is classified as physically active, with higher activity levels reported among males and individuals aged 30–39. However, a notable portion of youth, particularly college students, remain physically inactive, which presents a significant public health concern. The World Health Organization (WHO) recommends that adults engage in at least 150–300 min of moderate-intensity or 75–150 min of vigorous-intensity aerobic activity each week (4). Nevertheless, a systematic review conducted by Bajuaifer and Alrashdi (5) found that only 27% of Saudi college students meet these guidelines. Barriers to physical activity include academic workloads, insufficient facilities, and sociocultural constraints, particularly for female students (5). Numerous studies indicate a positive correlation between physical activity and GPA, suggesting that increased physical engagement may lead to enhanced academic performance and reduced psychological distress (6, 7).

Depression is one of the most prevalent mental health disorders impacting university students both globally and in Saudi Arabia (8). National estimates suggest a lifetime prevalence of Major Depressive Disorder (MDD) at 6.0% (9). Within university populations, the rates are even more alarming: at King Saud University, over 80% of students reported experiencing depressive symptoms, with approximately 40% categorized as experiencing severe to extremely severe levels (8). While some studies have found no direct correlation between GPA and depression, others indicate that students with higher GPAs tend to report fewer depressive symptoms (7–10). These varying findings highlight the necessity for more nuanced investigations, especially those that explore depression in conjunction with other behavioral and lifestyle factors.

Eating disorders (EDs) represent a significant yet often underrecognized factor impacting academic performance and overall well-being (11, 12), characterized by unhealthy obsessions with food, weight, and body image. EDs disproportionately affect young adults, particularly females (13). A study conducted at King Abdulaziz University revealed that 34% of students were at risk of developing an eating disorder, with females being twice as likely as males to be affected (14). Similarly, a study indicated a 28.7% prevalence of high-risk eating disorders among students at Taibah University, showing significant correlations with gender, age, BMI, and academic performance (15).

A significant amount of research has investigated the individual impacts of physical activity, depression, and eating disorders on academic performance. However, no studies have yet examined their combined influence within the context of higher education in Saudi Arabia. Additionally, much of the existing research has primarily centered on medical students, which restricts the applicability of the findings to a wider range of student populations across various academic disciplines. This absence of integrative, multidisciplinary research represents a critical gap in the literature.

This study is situated within the Saudi higher education context, where sociocultural factors may influence health behaviors and academic outcomes. Understanding these interconnections could lead to more holistic strategies for academic support, health promotion, and mental health services within Saudi higher education. To address this gap, the current study aims to examine the interconnected effects of physical activity, depressive symptoms, and eating disorder risk on academic performance among Saudi university students. By analyzing these variables within a comprehensive framework, this research seeks to enhance our understanding of how lifestyle and mental health factors interact to influence students’ academic success.

The specific objectives of the study are to: (1) assess the level and severity of depression, physical inactivity, and eating disorder—related symptoms among university students in the Kingdom of Saudi Arabia; (2) investigate the individual relationships between each of these factors and GPA; and (3) explore the combined associations of these variables on academic performance. This investigation may provide valuable insights for higher education stakeholders, policymakers, and campus health professionals aiming to promote student well-being and optimize academic achievement.

## 2 Materials and methods

### 2.1 Study design and sampling

This cross-sectional study was conducted from December 2024 to June 2025, using a non-probability convenience sampling approach, targeting students enrolled in undergraduate and postgraduate programs at universities across the Kingdom of Saudi Arabia. The inclusion criteria mandated that participants be currently enrolled in a Saudi university at any academic level. Participants were also required to be aged 18 years or older and able to understand and complete an English-language questionnaire. To recruit participants, a non-probability convenience sampling technique was employed. The minimum required sample size was calculated using OpenEpi, based on a 95% confidence level, a 5% margin of error, and an expected population proportion of 50%. This calculation established a target sample of at least 384 participants.

### 2.2 Data collection instrument

Data were gathered using a structured, self-administered online questionnaire, which was developed and distributed via the SurveyMonkey platform. The survey link was disseminated through informal digital channels commonly used by university students, including student social media platforms and peer-to-peer sharing across multiple universities. Participation was voluntary, and no incentives were provided. The questionnaire was available in English and designed for completion in approximately 10–15 min. It included an informed consent section followed by items that covered socio-demographic information and standardized scales. The demographic variables collected comprised age, gender, body mass index (BMI), smoking status, household income, academic level, and academic specialty. Following the demographic section, the questionnaire integrated validated tools to evaluate the primary study variables, including academic performance (GPA), physical activity (IPAQ-SF), depression symptoms (PHQ-9), and eating disorder symptoms (EDE-QS). Detailed descriptions of each of these instruments can be found in the sections below.

### 2.3 Academic performance

Academic performance was evaluated through students’ self-reported Grade Point Average (GPA). To maintain consistency in analysis, all reported GPAs were converted to a standardized 5.0 grading scale. For participants who initially reported their GPA using different systems (e.g., out of 4 or out of 100), values were proportionally converted to correspond with the 5-point system. For analytical purposes, GPA was utilized both as a continuous variable and as a binary categorical variable. In the binary classification, a cut-off of 3.75 was established, with students categorized as having high academic performance (≥3.75) or low academic performance (<3.75). This threshold is consistent with common standards for academic excellence and honors classifications in Saudi universities.

### 2.4 Physical activity level

Physical activity was evaluated using the International Physical Activity Questionnaire–Short Form (IPAQ-SF), a self-reported tool comprising nine items designed to capture physical activity over the preceding 7 days (16). The questionnaire gathers information on the frequency (days per week) and duration (minutes per day) of walking, moderate-intensity, and vigorous-intensity physical activities. For each activity type, a MET (Metabolic Equivalent of Task) score was calculated by multiplying the number of minutes, days, and assigned MET values (8.0 for vigorous activity, 4.0 for moderate activity, and 3.3 for walking). A total MET score was derived by summing the scores from these three domains. According to Craig et al. (17) the official IPAQ scoring protocol, participants were classified into three physical activity levels: Low (not meeting the criteria for moderate or high), Moderate (at least 600 MET minutes per week or moderate activity on five or more days), and High (vigorous activity on three or more days accumulating at least 1,500 MET-minutes per week, or total activity of at least 3,000 MET-minutes per week). The IPAQ-SF has been validated for use across international populations and exhibits acceptable reliability and validity for monitoring physical activity levels (17). The IPAQ-SF has also been used in Saudi populations, including studies involving health professionals and university-affiliated samples, supporting its applicability within the Saudi cultural context (18, 19).

### 2.5 Depression level

Depression symptoms were evaluated using the Patient Health Questionnaire-9 (PHQ-9), a rigorously validated self-report tool based on DSM-IV criteria (20).

The questionnaire comprises nine items, each scored on a 3-point scale ranging from 0 (“not at all”) to 3 (“nearly every day”), resulting in a total score that can vary from 0 to 27. Depression severity is categorized according to standard thresholds: minimal (0–4), mild (5–9), moderate (10–14), moderately severe (15–19), and severe (20–27) (20). For analytical purposes, a binary classification was also implemented, using a cut-off score of ≥10 to identify participants with probable clinically significant depression. This threshold has been validated in multiple studies and has been shown to yield optimal diagnostic performance, with pooled sensitivity and specificity values around 85% (20, 21). The PHQ-9 has been previously used in Saudi university and healthcare settings, including English-language administration, which supports its use in this study (22).

### 2.6 Eating disorders assessment

Eating disorder symptoms were assessed using the Eating Disorder Examination Questionnaire—Short Form (EDE-QS), which consists of 12 self-report items adapted from the original EDE-Q developed by Fairburn and Beglin (23). The EDE-QS aims to evaluate the key behavioral and cognitive features of eating disorders experienced over the past 7 days, serving as a concise yet psychometrically robust tool suitable for both clinical practice and research settings. Each item is rated on a 4-point Likert scale from 0 to 3, with higher scores reflecting greater severity. The total score can range from 0 to 36 (24).

For analytical purposes, the scale was divided into two domains: behavioral symptoms (items 1–10) and cognitive symptoms (items 11 and 12). Separate cumulative scores were computed for each domain, along with the overall total score, to provide a more nuanced understanding of the participants’ symptom patterns.

### 2.7 Ethical consideration

This study was conducted in accordance with the ethical standards established in the Declaration of Helsinki. Ethical approval was obtained from the Institutional Review Board (IRB) of Princess Nourah Bint Abdulrahman University (PNU) in Riyadh, Saudi Arabia [24-0766] [HAP-01-R-059]. Prior to participation, all respondents received an electronic informed consent form integrated within the online questionnaire. This consent form clearly outlined the purpose of the study, the voluntary nature of participation, the confidentiality of data, and the participants’ right to withdraw at any time without penalty.

### 2.8 Statistical analysis

All statistical analyses were conducted using IBM SPSS Statistics Version 31.0 (IBM Corp., Armonk, NY). The Shapiro–Wilk test was employed to assess the normality of continuous variables, revealing that most variables deviated from a normal distribution. Consequently, continuous variables were summarized using medians and inter-quartile ranges (IQRs), while categorical variables were presented as frequencies and percentages. Internal consistency of the EDE-QS and PHQ-9 in the present sample was assessed using Cronbach’s alpha. Analyses were conducted using an available-case approach (listwise deletion); therefore, sample sizes varied across analyses due to missing responses on specific variables, and participants with missing data were excluded only from the corresponding analysis.

Prior to conducting multivariate analysis, bivariate tests were carried out to explore associations between academic performance and potential predictors. Academic performance was initially categorized as a binary variable (e.g., high vs. low GPA). Chi-square (*χ*^2^) tests were utilized to examine relationships between binary or categorical predictors (e.g., gender, smoking status, income group, physical activity level, and academic level) and binary GPA. For continuous or ordinal predictors (e.g., age, depression score, and eating disorder score), eta (*η*) coefficients were calculated to evaluate the strength of their association with GPA. Following Cohen’s (2013) criteria, eta values were classified as minimal (<0.10), weak (0.10–0.29), moderate (0.30–0.49), or strong (≥0.50).

Only variables that demonstrated statistically significant associations in chi-square tests or at least weak associations via eta coefficients (*η* ≥ 0.10) were retained for the multivariable model consistent with a purposeful variable selection approach. A multiple linear regression analysis was subsequently conducted using continuous GPA as the dependent variable, based on the assumption of normal distribution of residuals. Regression results are reported using unstandardized coefficients (*B*) for interpretation, with standardized coefficients (*β*) provided for comparative purposes. Decisions regarding variable selection and modelling were guided by established best practices (25, 26). Given that predictors were selected following initial bivariate screening, *a priori* power analysis was conducted to assess whether the achieved sample size was sufficient for a multiple linear regression model of comparable complexity. Using G*Power (*F* tests; linear multiple regression: fixed model, *R*^2^ deviation from zero), assuming six predictors, a small-to-moderate effect size (*f*^2^ = 0.05), *α* = 0.05, and 80% power, the required sample size was approximately 286 participants. The analytic sample exceeded this requirement.

## 3 Results

### 3.1 Sociodemographic characteristics

A total of 400 students participated in this study, with a median age of 21 years, with an IQR of 3 years (19.50–22.50). Table 1 outlines the sociodemographic characteristics of the study participants (*N* = 400). The sample comprised predominantly females (79.8%). More than half of the participants (52.4%) fell within the normal BMI range, while the remaining individuals were categorized as underweight, overweight, or obese. A significant majority were non-smokers. Regarding sleep patterns, over half of the participants (51.5%) reported sleeping 7 or more hours per night. Regionally, the central area was the most represented (44.3%), followed by the eastern and western regions. Most participants (68%) indicated an annual family income of less than 100,000 SAR. Additionally, more than half (52.5%) received a monthly allowance of less than 2,000 SAR, and the majority were unemployed (82.5%).

**Table 1:** Sociodemographic characteristics.

Variable	Category	Frequency (%)
Gender	Male	81 (20.3%)
	Female	319 (79.8)
*BMI classification	Underweight (<18.5)	58 (14.9)
	Normal (18.5–24.9)	204 (52.4%)
	Overweight (25–29.9)	81 (20.8%)
	Obese (≥30)	46 (11.8%)
Smoking status	Not smoking	361 (90.3%)
	Current/former smoker	39 (9.8%)
Average night sleep	Less than 5 h	45 (11.3%)
	5–6 h	149 (37.3%)
	7 h or more	206 (51.5%)
Region	Central	177 (44.3%)
	Northern	18 (4.5%)
	Southern	40 (10%)
	Eastern	107 (26.8%)
	Western	58 (14.5%)
Annual family income	Less than 100,000	272 (68%)
	100,000–200,000	69 (17.3%)
	More than 200,00	59 (14.8%)
Monthly allowance	No allowance	81 (20.3%)
	Less than 2000	210 (52.5%)
	2000 or more	109 (27.3%)
Working status	Not employed	330 (82.5%)
	Employed	70 (17.5%)

BMI (body mass index *N = 389).

### 3.2 Academic performance and educational characteristics

The median GPA was noted at 4.5, with an IQR of 0.70 (4.15–4.85). Table 2 displays the descriptive statistics concerning the educational characteristics of the participants. A significant majority of participants (87.5%) were classified as high academic performers, indicated by a GPA of 3.75 or higher. Most of the participants were undergraduate students, comprising 91.2% of the sample. Additionally, over half of the participants (53.8%) were enrolled in health-related colleges, followed by 32.2% in scientific disciplines and 14% in humanitarian fields. In terms of the level of study, 37.5% of participants were in their third or fourth year, 35% were in their first or second year, and 27.5% had completed five or more years of study.

**Table 2:** Descriptive statistics of educational characteristics.

Variable	Category	Frequency (%)
GPA category *N* = 392	High (3.75/5 or more)	343 (87.5%)
	Low (less than 3.75/5)	49 (12.5%)
Type of study *N* = 398	Undergraduate	363 (91.2%)
	Postgraduate	35 (8.8%)
Specific category *N* = 394	Health colleges	212 (53.8%)
	Scientific colleges	127 (32.2%)
	Humanitarian colleges	55 (14%)
Level of study (years) *N* = 400	1–2	140 (35%)
	3–4	150 (37.5%)
	5 and more	110 (27.5%)

### 3.3 International Physical Activity Questionnaire–Short Form (IPAQ-SF)

**Figure 1:**
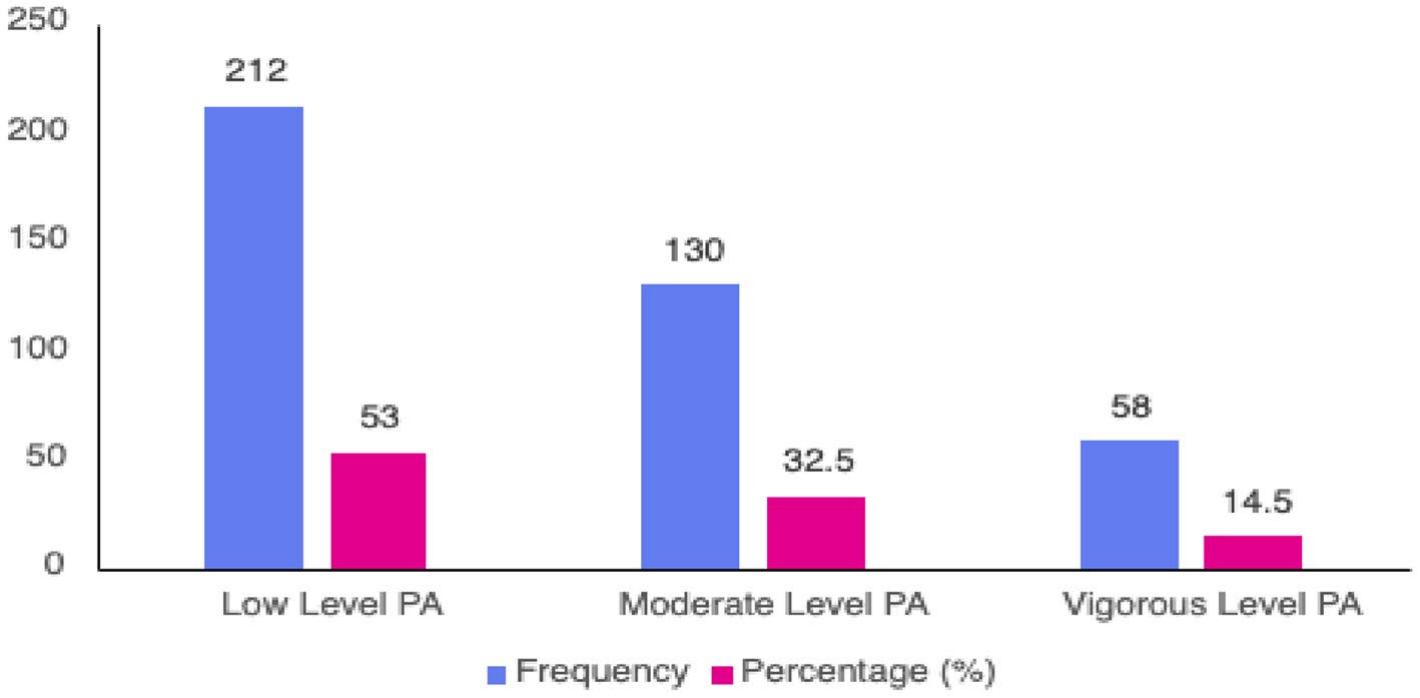
Physical activity profile of participants based on IPAQ-SF (*N* = 400).

Figure 1 shows the Physical Activity Profile of Participants Based on IPAQ-SF classification (*N* = 400). According to the IPAQ-SF classification criteria, more than half of the participants (53%) were classified as having a low level of physical activity. Meanwhile, 32.5% engaged in moderate activity, and only 14.5% were identified as participating in vigorous physical activity. Regarding compliance with the World Health Organization (WHO) recommendations for physical activity, 47% of participants met the minimum levels for health benefits, while 53% did not.

### 3.4 Depression level and eating disorder

The internal consistency of the study instruments was evaluated using Cronbach’s alpha. The EDE-QS demonstrated excellent internal consistency (Cronbach’s *α* = 0.90). PHQ-9 showed good internal consistency (Cronbach’s *α* = 0.85).

Table 3 provides descriptive statistics and classifications regarding eating disorder symptoms and depression within the study sample. The median total score on the EDE-QS was 12, with an IQR of 14 (5–19). Subscale analysis indicated a median behavioral score of 9 with an IQR of 11 (3.5–14.5) and a cognitive score of 2 with an IQR of 3 (0.5–3.5). The median total score on the PHQ-9 was 10 with an IQR of 9 (6–15), reflecting a significant presence of depressive symptoms among participants. When categorized according to PHQ-9 severity thresholds, the most common classification was moderate depression, noted in 29.3% of participants, followed by mild (26.0%) and minimal (23.3%) symptoms. Moderately severe and severe depression were reported by 13.8 and 7.8% of participants, respectively. According to established clinical cutoffs for the PHQ-9, 50.7% of the sample fell into the category of significant depression, while 49.3% were classified as having non-significant depression.

**Table 3:** Descriptive statistics and classifications regarding eating disorder scores and depression.

Variable	Median	IQR^a^
Eating disorder score	12	14 (5–19)
Eating disorder behavioral score	9	11 (3.5–14.5)
Eating disorder cognitive score	2	3 (0.5–3.5)
Depression score	10	9 (6–15)
Variable	Category	Frequency (%)
Depression level	Minimal	93 (23.3%)
	Mild	104 (26%)
	Moderate	117 (29.3%)
	Moderately sever	55 (13.8%)
	Sever	31 (7.8%)
Depression classification	Non-significant depression	197 (49.3%)
	Significant depression	203 (50.7%)

a IQR, interquartile range.

### 3.5 Sociodemographic, lifestyle, and psychological factors associated with academic performance

Table 4 illustrates the relationships between various sociodemographic and lifestyle factors and academic performance, categorized as high versus low GPA, utilizing Chi-square tests. Among all the variables assessed, specialty category demonstrated a statistically significant association with academic performance (*χ*^2^ = 17.336, df = 2, *p* < 0.001, Cramér’s V = 0.211), indicating a moderate effect size. Furthermore, adherence to physical activity guidelines was significantly linked to academic performance (*χ*^2^ = 6.178, df = 1, *p* = 0.013, Cramér’s V = 0.126), suggesting a small to moderate effect. Notably, students who complied with physical activity guidelines were over twice as likely to achieve a high GPA (≥3.75) compared to those who did not (OR = 2.23, 95% CI: 1.17–4.24). Other variables, such as gender, BMI category, smoking status, average nightly sleep, income, working status, and level of study, did not indicate statistically significant associations (*p* > 0.050). Effect size coefficients revealed a mild association between academic performance and age (*η* = 0.220), depression score (*η* = 0.271), and eating behavior score (*η* = 0.274), with a minimal association observed for eating cognitive score (*η* = 0.069).

**Table 4:** Association between academic performance and selected sociodemographic and lifestyle variables.

Variable	Academic performance (high vs. low)			
	*χ* ^2^	Df	*p*-value	Cramer V
Gender	2.297	1	0.130	0.077
BMI classification	1.953	3	0.582	0.071
Smoking status	3.108	1	0.078	0.089
Average night sleep	2.394	2	0.302	0.078
Annual family income	0.113	2	0.945	0.017
Monthly allowance	3.917	2	0.141	0.100
Working status	1.134	1	0.287	0.054
Type of study	0.297	1	0.586	0.028
Specialty category	17.336	2	< 0.001*	0.211
Level of study (years)	2.888	2	0.236	0.086
Compliance with PA	6.178	1	0.013*	0.126

BMI = body mass index; PA = physical activity; ^*^Cramer V = effect size for chi-square tests; Df = degree of freedom; *χ*^2^ = chi-square test statistics; *p*, probability value, ^*^ = statistically significant (*p* < 0.05).

### 3.6 Multivariable analysis of physical, psychological, and academic predictors of academic performance

A multiple linear regression analysis was performed to investigate the predictive effects of physical activity compliance, cognitive and behavioral symptoms of eating disorders, depression, age, and academic specialty (medical vs. non-medical) on academic performance, as measured by GPA (see Table 5). The overall model demonstrated statistical significance, *F*(6, 381) = 4.114, *p* < 0.001, though it accounted for a modest portion of the variance in GPA (*R*^2^ = 0.061, Adjusted *R*^2^ = 0.046).

**Table 5:** Multiple linear regression predicting academic performance.

Predictor	*B*	SE	*β*	95% CI	*p*-value
Age (years)	0.006	0.008	0.041	[−0.009, 0.022]	0.423
Compliance with physical activity vs. noncompliance	0.137	0.055	0.125	[0.029, 0.245]	0.013
Medical specialty (vs. non-medical)	0.179	0.056	0.163	[0.070, 0.288]	0.001
Eating disorder—cognitive score	0.025	0.020	0.085	[−0.013, 0.063]	0.202
Eating disorder—behavioral score	−0.011	0.005	−0.134	[−0.021, −0.001]	0.035
Depression score (PHQ-9)	−0.001	0.005	−0.008	[−0.010, 0.009]	0.882

*B* = regression coefficient, SE = standard error, *β* = beta coefficient, 95% CI = 95% confidence interval, *p* = probability value.

Among the various predictors, enrolment in a medical specialty (*B* = 0.179, *p* = 0.001) and adherence to the recommended level of physical activity (*B* = 0.137, *p* = 0.013) were found to significantly predict higher GPAs. Conversely, higher levels of behavioral symptoms associated with eating disorders correlated with lower GPAs (*B* = −0.011, *p* = 0.035). In contrast, cognitive symptoms of eating disorders (*B* = 0.025, *p* = 0.202), depression scores (*B* = −0.001, *p* = 0.882), and age (*B* = 0.006, *p* = 0.423) did not emerge as significant predictors. While the model achieved overall significance, these findings indicate that only a few specific factors—namely, academic specialty, engagement in physical activity, and disordered eating behavior—significantly influence academic performance in this sample.

## 4 Discussion

This study aimed to investigate the relationships between physical activity, depression, and eating disorder symptoms and their impact on academic performance among Saudi university students. The findings offer valuable insights into the behavioral and psychological factors that influence GPA, highlighting both risk and protective elements associated with academic success in this demographic. The results revealed a significant correlation between higher GPA and achieving recommended levels of physical activity. Conversely, academic performance was negatively impacted by elevated levels of disordered eating behaviors. Notably, despite a considerable portion of the sample reporting moderate to severe depressive symptoms, depression scores did not significantly predict academic achievement.

### 4.1 Physical activity and academic performance

In line with existing literature, the findings reveal a notable positive correlation between adherence to WHO-recommended physical activity levels and enhanced academic performance. Students who complied with the physical activity guidelines were over twice as likely to achieve a high GPA compared to their peers who did not, even after adjusting for various factors. This adds to the growing evidence that regular physical activity is associated with cognitive functions such as executive functioning, attention, and memory, which may be relevant to academic performance (27, 28). This underscores the positive impact of physical activity on academic outcomes in Saudi Arabian universities, emphasizing its essential role and strong association.

Interestingly, more than half of the participants were found to have low levels of physical activity, mirroring national and regional trends noted in previous studies involving Saudi students (3, 5, 29, 30). This underscores the significant role that physical activity plays as a predictor of academic success. Despite the overall low rates of activity, the findings suggest that even modest increases in physical engagement could lead to considerable improvements in academic performance. These results emphasize the need for structured interventions, such as incorporating exercise programs into university life or academic curricula, to foster a more active student population and enhance cognitive health.

### 4.2 Eating disorder symptoms and academic outcomes

Disordered eating behaviors were frequently reported within the study population, with a substantial proportion of the participants demonstrating elevated behavioral symptom scores on the EDE-QS. Regression analysis revealed that behavioral symptoms of eating disorders—such as restrictive eating, meal skipping, and emotional eating—were inversely related to GPA. This finding aligns with existing research that links disordered eating to impaired concentration, decreased energy levels, and increased psychological distress (11, 12).

Interestingly, cognitive symptoms, such as body image concerns and weight pre-occupation, did not significantly predict GPA. This may suggest that while cognitive preoccupations can be psychologically burdensome, they do not always result in functional impairments unless they are expressed behaviorally. The distinction between cognitive and behavioral symptoms warrants further investigation and could be clinically relevant for the development of targeted wellness programs at universities. Furthermore, the regression model implies that untreated disordered eating behaviors—rather than only clinical eating disorders—negatively impact academic performance, reinforcing the need for early screening and preventive services on college campuses.

### 4.3 Depression: high prevalence, limited predictive power

In this study, over half of the participants (50.7%) reported clinically significant depressive symptoms based on PHQ-9 scores, with moderate depression identified as the most prevalent severity level (29.3%). These findings align with previous research conducted in Saudi Arabia. For instance, a study found that 48.8% of university students experienced moderate to severe depressive symptoms using the same PHQ-9 scale (31). Likewise, a cross-sectional study at King Saud University reported a 44% prevalence of mood disorders, noting higher rates among non-health students compared to their counterparts in health-related programs (8).

Despite the high prevalence of depressive symptoms observed in the current sample, depression scores did not significantly predict academic performance. This outcome contrasts with several earlier studies that have associated depression with lower GPA (7, 32), yet it is consistent with others that demonstrate inconsistent or mediated relationships (8). One potential explanation could be the multifaceted nature of depression, where symptoms such as fatigue and hopelessness do not always lead to academic failure, especially among high-functioning students who may possess resilience or benefit from structured academic environments.

Additionally, the cross-sectional design may have limited the ability to discern causal or time-dependent effects. Depression could have a delayed impact on academic outcomes, or its influence might be mitigated by other variables, such as social support, faculty engagement, or coping mechanisms that were not measured in this study. Future longitudinal or mixed-methods research could provide greater insight into these dynamics.

### 4.4 Sociodemographic and contextual factors

In this study, various sociodemographic factors—including gender, BMI, smoking habits, sleep quality, income, employment status, and year of study—did not demonstrate significant connections with academic performance. These results imply that while such variables may influence students’ lifestyle or health behaviors, they are not robust indicators of academic achievement. Regarding age and psychological predictors like depression and eating disorders, only minimal to mild associations were observed in relation to academic performance, and these were not statistically significant. This indicates that their effect on GPA is limited or possibly mediated by other factors.

The findings suggest that academic discipline and physical activity are more strongly associated with mental health and academic outcomes compared to demographic variables or health habits, and may represent relevant areas for future investigation or intervention, emphasizing their advantageous role in relation to psychological well-being and academic success. A notable exception was found concerning the students’ academic specialty; those enrolled in medical or health-related colleges tended to achieve higher GPAs. This trend may reflect the competitive nature of medical education in Saudi Arabia, where students are often highly motivated, better supported, and more academically engaged. Alternatively, variations in grading systems or faculty expectations among different colleges might account for some of this variance. These results underscore the importance of considering the academic environment and program-specific factors when interpreting GPA outcomes.

### 4.5 Multivariable analysis: integrated predictors of academic success

The study aimed to examine the predictive effects of physical activity and psychological factors on academic performance. A multivariable analysis was performed using multiple linear regression, which revealed that among the predictors (age, eating disorders, depression, physical activity, and academic specialty), three key factors significantly influenced academic success: academic specialty, physical activity, and behavioral symptoms of eating disorders.

Although the regression model explained only a modest 6.1% of the variance in GPA, it suggested that academic success is influenced not only by intellectual ability but also by lifestyle and health-related behaviors, consistent with existing literature (33). In contrast, a separate 2020 study found that light physical activity negatively correlated with GPA in males, while increased sitting time was linked to a higher GPA among men. However, this study also reported a positive correlation between physical activity and GPA in female participants (34). Additionally, another study found an insignificant correlation between GPA and physical activity among health science students (35). These conflicting results highlight the complexity of the relationship between physical activity and academic performance in college students, which may vary by gender and the type of physical activity (34).

Interestingly, students receiving treatment for anorexia or bulimia were found to have higher GPAs (11), while disturbances related to eating and body image were associated with lower GPAs (12). These findings indicate that treated eating disorders may positively impact academic performance, whereas untreated eating disorders can have negative effects. Furthermore, physical activity does not solely determine GPA; the behavioral symptoms of eating disorders are also influenced by various factors, including treatment status and specific eating disorder symptoms.

Notably, age, depression, and cognitive symptoms related to eating were not significant predictors, underscoring the multifactorial nature of academic performance. The relatively low explanatory power of the model indicates a need to consider additional variables—such as motivation, sleep quality, time management, stress resilience, and digital media use—in future studies.

### 4.6 Implications

The results underscore potentially actionable areas for university health and academic support services. While causal or preventive conclusions cannot be drawn from this cross-sectional study, promoting regular physical activity through accessible gym facilities, peer-led exercise groups, or wellness breaks integrated into the curriculum may be considered as supportive strategies to foster both academic and psychological well-being. Additionally, implementing routine screening programs for disordered eating and depressive symptoms—alongside low-barrier counselling and nutrition services—could help identify students at risk and inform supportive interventions that may mitigate factors associated with poorer academic performance. Importantly, these implications should be interpreted cautiously and viewed as exploratory, given the study design and sample characteristics. Overall, the findings highlight the value of an interdisciplinary approach that aligns health promotion with educational success, where collaboration among faculty members, student affairs professionals, and campus health providers may contribute to creating a learning environment in which physical and mental well-being are recognized as important correlates of academic excellence.

### 4.7 Limitation

This study employed a cross-sectional design, which constrains the ability to infer causal relationships between the examined variables and academic performance. Future research should adopt longitudinal designs to gain a clearer understanding of the direction and stability of these relationships over time. Moreover, the limited generalizability of the findings is noteworthy, as the sample was predominantly female and therefore, the findings should be interpreted as exploratory rather than representative of all Saudi university students. Future studies should strive for a more diverse and balanced representation in terms of gender distribution. In addition, the study relied on self-reported measures, which may be subject to recall bias and social desirability effects. Although English proficiency was required for participation, it was not formally assessed and may limit generalizability of the findings. Although standardized instruments were used, cultural factors such as mental health stigma, lifestyle expectations, and sociocultural norms specific to the Saudi context were not directly measured. Furthermore, while the employed instruments have been used in Saudi university and healthcare populations, formal cultural adaptation procedures were not undertaken in the present study and should be considered in future research.

## 5 Conclusion

This study presents significant evidence that physical activity and disordered eating behaviors serve as important predictors of academic performance among Saudi university students. In contrast, while depressive symptoms are common, they do not independently predict GPA. These findings suggest a necessary shift in how institutions approach student success, emphasizing the importance of addressing behavioral health and lifestyle factors within comprehensive academic support strategies. Future research utilizing longitudinal designs and a broader range of psychosocial variables will be crucial in developing interventions that promote resilience, wellness, and academic achievement across diverse student populations.

## Data Availability

The raw data supporting the conclusions of this article will be made available by the authors, without undue reservation.
