# Supplementary figures and images for: Associations of physical activity, disordered eating, and depressive symptoms with academic performance among Saudi university students
Source: Front Public Health. 2026 Feb 12;14:1769363. doi: 10.3389/fpubh.2026.1769363 (PMC12935905; doi:10.3389/fpubh.2026.1769363)

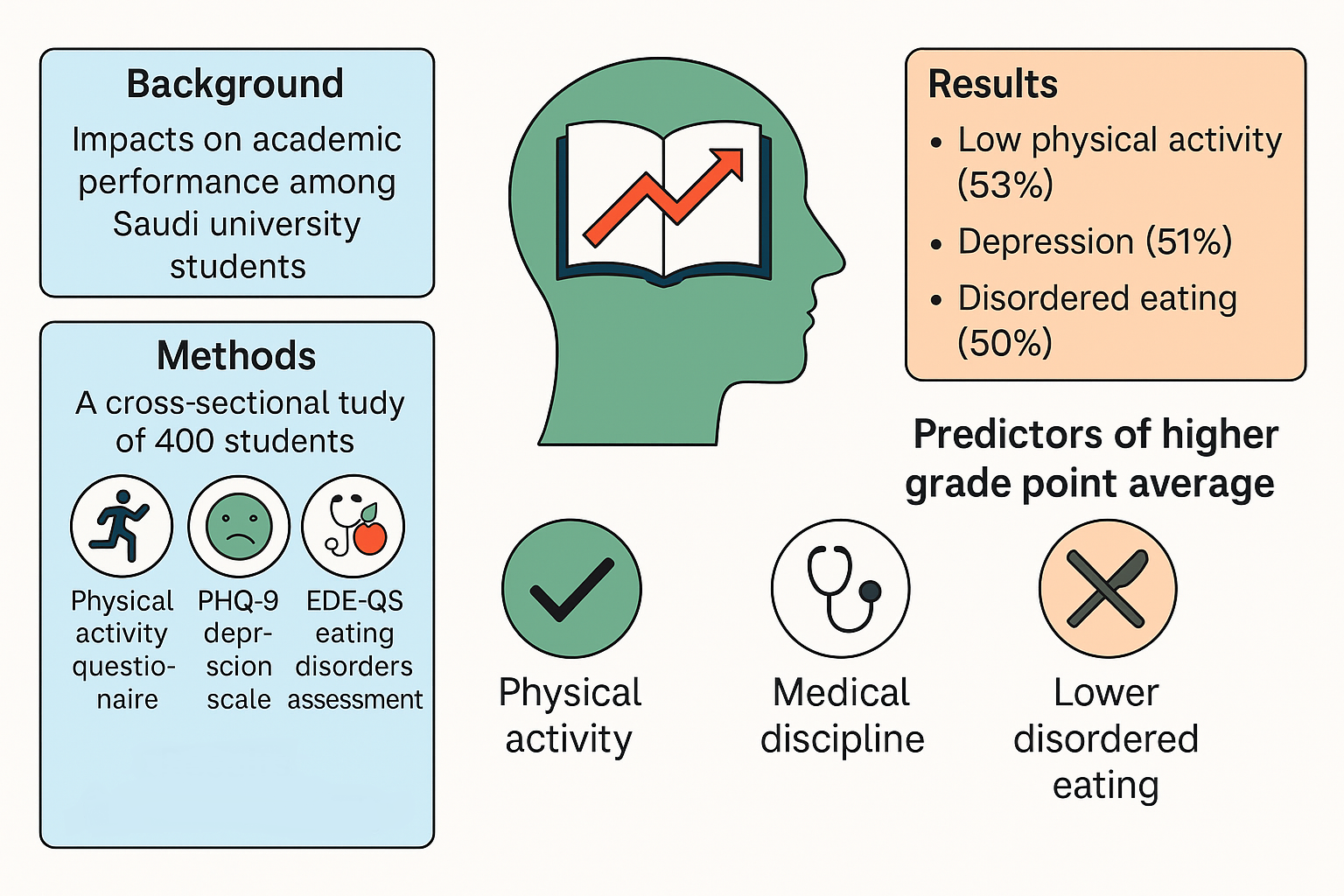

Supplement: Supplementary file 1 [file Image_1.PNG]
